# Whole‐Brain Diffusion MRI at 7T With Universal Multiband GRAPE pTx Pulses

**DOI:** 10.1002/mrm.70588

**Published:** 2026-09-14

**Authors:** Marten Laufenberg, Daniel Löwen, Mónica Ferreira, Nicolas Boulant, Jennifer Faber, Tony Stöcker

**Affiliations:** ^1^ German Center for Neurodegenerative Diseases (DZNE) Bonn Germany; ^2^ Université Paris‐Saclay, Commissariat à l'Energie Atomique, CNRS NeuroSpin, BAOBAB Gif sur Yvette France; ^3^ Department of Parkinson's Disease, Sleep and Movement Disorders, Center of Neurology University Hospital Bonn Bonn Germany; ^4^ Department of Neuroradiology University Hospital Bonn Bonn Germany; ^5^ Department of Physics & Astronomy University of Bonn Bonn Germany

**Keywords:** diffusion MRI, parallel transmission, ultra‐high field

## Abstract

**Purpose:**

To improve signal strength and homogeneity of whole‐brain diffusion‐weighted imaging (DWI) at 7T.

**Methods:**

Universal multiband excitation and refocusing pulses were optimized with the gradient ascent pulse engineering (GRAPE) algorithm across a database of $B_{1}^{+}$ and $B_{0}$ maps obtained from 20 subjects. Slice‐selective multiband pulses were optimized in a two‐step optimization for transverse and sagittal slice orientations. The optimized pTx pulses were compared to standard circular polarized (CP) pulses in five subjects using a multiband diffusion‐weighted EPI sequence at 1.5 mm isotropic resolution and in one subject at 1.05 mm resolution. The evaluation included Bloch simulations, flip angle mapping, SNR analysis, and diffusion model fitting (DTI and NODDI).

**Results:**

Both GRAPE pulse sets with transverse and sagittal slice orientation improved signal homogeneity and SNR compared to CP pulses across all subjects and head sizes, with particularly high gains in the cerebellum. Sagittal GRAPE pulses yielded the highest mean signal and lowest standard deviation across the brain, while transverse GRAPE pulses provided better cerebellar signal at the cost of signal loss in off‐resonant frontal and temporal regions of large heads. The improved signal translated into more reliable DTI and NODDI fitting, especially in the cerebellum.

**Conclusion:**

Universal multiband GRAPE pTx pulses substantially improve signal homogeneity and SNR in whole‐brain DWI at 7T, enabling more reliable diffusion microstructure mapping, particularly in the cerebellum.

## 1 Introduction

Whole‐brain diffusion‐weighted imaging (DWI) at ultra‐high field (7T and above) is hampered by inhomogeneous $B_{1}^{+}$ fields leading to signal dropouts and reduced SNR in large parts of the brain. This is one of the main reasons why most large‐scale diffusion studies to date have been conducted at lower field strengths. Although, MRI scanners with high‐performance gradient systems for DWI have initially been developed for 3T [1], such gradient systems become increasingly available at 7T [2]. Despite the challenges of increased $B_{1}^{+}$ and $B_{0}$ inhomogeneity and shortened $T_{2}$ and $T_{2}^{*}$ values, 7T holds great potential for whole‐brain DWI due to generally higher SNR and achievable resolution [2, 3, 4]. The challenge of inhomogeneous $B_{1}^{+}$ fields is typically addressed using parallel transmission (pTx) techniques, which allow tailoring the RF pulse shapes to the individual subject's $B_{1}^{+}$ and $B_{0}$ field distribution. These field distributions are usually obtained in calibration prescans, which adds extra scan time to the examination and requires fast pTx pulse design algorithms, as the pulses are optimized during the examination.

In universal pulse design [5] pTx pulses are optimized across a database of $B_{1}^{+}$ and $B_{0}$ maps from multiple subjects rather than on individual subjects. This eliminates the need for subject‐specific calibration scans and enables offline pulse design. Nonselective universal pTx pulses have been shown to improve signal homogeneity in the brain, while being robust against inter‐subject variability in several applications at 7T [6, 7]. Common DWI sequences use multiband spatial‐selective pulses rather than nonselective pulses, because spatial‐selective pulses enable single‐shot k‐space acquisitions, avoiding shot‐to‐shot phase variations caused by motion during the diffusion gradients. This complicates RF pulse design, raises sequence programming difficulties and presents apparent challenges regarding the design of universal pTx pulses due to the variability in slice position and orientation between subjects. Universal slice‐selective single‐ and multiband pulses have been applied in fMRI and GRE sequences [8, 9], but their application in DWI has not been demonstrated yet. The design of multiband pTx pulses for DWI is rather challenging due to their high power and specific absorption rate (SAR) requirements especially at high flip angles and high multiband factors typically used in spin‐echo DWI.

Initial results obtained with RF shimming and kT‐spokes pTx pulses used for whole‐brain DWI showed promising improvements in SNR and homogeneity [10, 11, 12]. However, the performance of kT‐spokes pulses is limited by the low number of degrees of freedom in the optimization and their sensitivity to off‐resonance effects [13]. More recently, the gradient ascent pulse engineering (GRAPE) algorithm [14, 15] was proposed for parameter‐free pTx pulse optimization with increased degrees of freedom yielding improved robustness to off‐resonances [14]. Pulse optimization with the GRAPE algorithm is time‐consuming and not practical during an examination, but combining it with the concept of universal pulses overcomes this limitation.

Recently, we showed that slice‐selective universal pulse design with the GRAPE algorithm is feasible by constraining the optimization of the pulse to a target bandwidth [16]. In this work, we apply this concept to the design of multiband excitation and refocusing pulses for whole‐brain DWI at 7T. We compare the performance of the optimized GRAPE pulses to standard circular polarized (CP) pulses in vivo in five subjects.

## 2 Methods

### 2.1 Pulse Design

The pTx pulse design for multiband excitation and refocusing pulses follows the second strategy for slice‐selective pTx pulse optimization from our previous work [16]. This strategy splits the pulse optimization into two steps: The optimization of a spectral‐selective pulse on a whole brain target and a second optimization step, where the pulses are refined for specific slice positions. The optimization was performed independently for excitation and refocusing pulses on a database of $B_{1}^{+}$ and $B_{0}$ maps from 20 subjects applying the concept of universal pulses [5]. The $B_{1}^{+}$ and $B_{0}$ maps in the database were acquired with a 3D dual refocusing echo acquisition mode (3DREAM) sequence [17] (4 mm iso) and a 3D dual‐echo GRE sequence (3 mm iso).

The optimization of excitation and refocusing pulses with the GRAPE algorithm [14, 15] was initialized with spectral‐selective sinc shapes with target bandwidths $\Delta f_{T}$. The target bandwidths and durations were set to $\Delta f_{T,exc}$ = 700 Hz, t = 6 ms for excitation and $\Delta f_{T,ref}$ = 350 Hz, t = 8 ms for refocusing pulses leading to time‐bandwidth products (TBP) of 4.2 and 2.8, respectively. These bandwidths fulfill the requirements of “fat unfocusing” [18] such that additional fat suppression pulses could be avoided.

In order to maintain the target bandwidth during the optimization, $B_{0}$‐maps in the database were duplicated five times and a random frequency shift $\Delta f(\vec{r})$ was added to each voxel following a Gaussian distribution with a standard deviation of 3.5 kHz. For the whole‐brain optimization target, a passband with target flip angle 90° (excitation) and 180° (refocusing) was defined as $\left|\Delta f(\vec{r})\right|\leq \Delta f_{T}/2$ and a stopband with target flip angle zero was defined as $\left|\Delta f(\vec{r})\right|>\Delta f_{T}/2$. The passband is centered around the frequency offset of the respective voxel as determined by the $B_{0}$ maps. The standard deviation of 3.5 kHz was empirically determined such that there are no side lobes present in the spectrum of the pulses over the frequency range in the brain caused by the application of the slice gradient. Such side lobes would lead to undesired excitation outside of the desired slice [16].

A complex least‐squares cost‐function with a flat target phase in the passband was used to achieve a constant phase in slice‐selection direction. The random frequency shifts cause a phase evolution, which corresponds to the effect of the slice selection gradient. This unwanted phase evolution is typically reversed by a slice rewinder gradient, which is not part of the optimization. Therefore, the phase evolution due to the random frequency shifts was removed by subtracting a free precession rotation from the Bloch simulation results of each voxel.

The maximum energy of the singleband pulses in the optimization was set to 260 mJ (excitation) and 500 mJ (refocusing) for a multiband factor MB = 2, which resulted in SAR values close to the limit of 10 W/kg (first‐level mode) over a 6 min time‐window for the sequence parameters given below. The voltage per channel was constrained to 95 V such that the maximum voltage of 190 V could not be exceeded for multiband pulses. The maximum slew rate was set to 190 T/m/s and the maximum gradient amplitude to 67 mT/m. The result of the first optimization step is a spectral‐selective pulse optimized for the whole brain, irrespective of the slice position.

In the second step, the RF shapes were further optimized for each slice by adding the weighting term exp(−d/10 mm) to the cost function, where d is the distance to the center of the current slice [16]. This weighting improves pulse performance in the respective slice while allowing for moderate variation in FOV position and orientation [8]. The second optimization step was performed for transverse and sagittal slice orientations. Although, oblique transverse slices reduce the number of required slices for whole‐brain coverage, we optimized purely transverse slices, as simulations indicated reduced pulse performance especially in lower slices in the oblique orientation. Pulses designed for sagittal slices were reported to have improved performance with fewer black holes in the brain compared to transverse orientations for the coil used [12]. For transverse GRAPE pulses the slice position of the first pulse was aligned with the top of the $B_{1}^{+}$ maps in the database, while for sagittal GRAPE pulses the slice positions were equally distributed around the center of the brain. The gradient shapes were not modified during the second optimization step for two reasons: Gradients within a multiband slice group must be identical, and varying gradients between slice groups can introduce discontinuities of the signal in adjacent slices [11, 19].

In order to reduce the computational load and the memory demand, all pulse optimizations were performed on a 40 μs time raster and only one pulse for every second slice was optimized such that the same pulse was used for two neighboring slices. After the optimization, a constant slice gradient corresponding to the target slice thickness was added and the pulses were superimposed to create multiband pulses. The target slice thickness was defined as the full width at half maximum (FWHM) of the simulated slice profile. The singleband pulses were linearly interpolated to a 10μs time raster to avoid discretization errors and wraps in frequency space. Frequency offsets were applied to both singleband pulses symmetrically around the center frequency. We optimized slice‐specific GRAPE pulses for 1.5 and 1.05 mm resolution. For 1.05 mm resolution an additional set of pulses for multiband factor three was optimized with lower maximum energy of the singleband base pulses.

The optimized GRAPE pulses were compared to standard Shinnar‐Le‐Roux (SLR) CP pulses provided by the vendor with a duration of 6 ms (excitation) and 14 ms (refocusing) and a bandwidth of 793 Hz (excitation) and 362 Hz (refocusing). Variable‐rate excitation (VERSE) was disabled to keep the bandwidth constant throughout the pulse such that the requirements of “fat unfocusing” could be fulfilled. CP pulse parameters were chosen such that SAR values were close to the limit of 20 W/kg (first‐level mode) at a reference voltage of 300 V.

### 2.2 Experiments

Data from five subjects (four male, one female, age 25–44, cf. Table S1), which were not part of the pulse design database, were acquired on a Siemens MAGNETOM 7T Plus scanner (Siemens Healthineers, Erlangen, Germany) using a 32RX/8TX RF head coil (Nova Medical, Wilmington, USA). All data were acquired in first‐level controlled SAR mode. All participants provided written informed consent in accordance with local ethics regulations.

The product single‐shot blipped controlled aliasing in parallel imaging (CAIPI) diffusion‐weighted EPI sequence [20] with GRAPPA (R = 2) and multiband (MB = 2) acceleration was modified to use either CP or pTx pulses. The nominal b values were set to $b\;=\;0\;{\text{s/mm}}^{2}$ (11 volumes), b = 1000 s/mm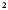 (30 directions) and b = 2000 s/mm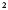 (70 directions). The directions were determined with an algorithm that ensures optimal angular coverage for multishell acquisitions [21]. The phase encoding direction was anterior‐posterior and three additional volumes without diffusion encoding were acquired with inverted phase encoding direction in a separate scan. For GRAPE pulses the orientation was either transverse (GRAPE TRA) or sagittal (GRAPE SAG), while for CP pulses oblique transverse slices were acquired in order to minimize the required number of slices for whole‐brain coverage. The imaging volume was aligned to the top of the brain for transverse GRAPE pulses and centered on the middle of the brain for sagittal GRAPE pulses. Images were acquired at 1.5 mm resolution with an in‐plane FOV of 210×210 mm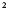 and a slice thickness of 1.5 mm. The partial Fourier (PF) factor in phase encoding direction was set to 6/8 and the readout bandwidth was 1786 Hz/px. This resulted in 102 slices (TR = 5.4 s, TA = 11:11 min) for both GRAPE acquisitions and 94 slices (TR = 5.0 s, TA = 10:24 min) for the CP acquisition. The echo time was minimized for all measurements resulting in TE = 66 ms for the GRAPE pulses and TE = 70 ms for the CP pulses. For the second subject two additional datasets were acquired at an isotropic resolution of 1.05 mm with multiband factors two (142 slices) and three (141 slices) and a reduced number of directions (b = 0 s/mm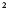: 7 volumes, b = 1000 s/mm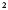: 20 directions, b = 2000 s/mm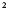: 45 directions) to account for the longer volume TR (MB2: TA = 10:56 min, TR = 8.5 s MB3: TA = 7:46 min, TR = 5.9 s). GRAPPA acceleration was set to R = 3 to allow for an echo time of TE = 77 ms. The CP acquisition in this experiment was done in pure transverse orientation.

The product EPI sequence acquires autocalibration signal (ACS) prescans for in‐plane GRAPPA (24 reference lines) and slice‐GRAPPA calibration with singleband pulses and a single‐shot EPI readout. Fat saturation was disabled for imaging scans, but enabled for ACS scans as this reduced artifacts in the images. We compared singleband GRAPE pulses optimized for the whole brain to CP pulses in ACS scans by acquiring data at all three b values for one subject, but used CP pulses in ACS scans for all other measurements. It is currently not possible to use individual pulse shapes for each slice in the ACS scans, as the number of pulse shapes allowed by the scanner is limited to 256.

Flip angle ratio maps of multiband refocusing pulses (CP and GRAPE) were acquired for subject five using a spin‐echo/stimulated‐echo (SE/STE) approach [22, 23, 24]. Data were acquired with an accelerated (R = 3) single‐shot spiral sequence implemented with Pulseq [25, 26]. For this purpose, a custom pTx extension was added to the Pulseq file format and the interpreter sequence was modified to support pTx pulses. A $90^{\circ }-2\alpha_{\text{nom}}-\alpha_{\text{nom}}$ nominal flip angle scheme with $\alpha_{\text{nom}}$ varying from 50° to 160° in 10° steps was used and the same CP and GRAPE multiband excitation and refocusing pulses as in the diffusion‐weighted EPI sequence were employed. The excitation pulse served as the first pulse, while the refocusing pulse served as the second and third pulse. The in‐plane resolution of the flip angle measurements was 4 mm, the spiral readout duration was 5.1 ms, the echo time was TE = 25 ms and the repetition time TR = 10.2 s for GRAPE and TR = 9.4 s for CP pulses. The total acquisition time was 3:15 min for the GRAPE pulses and 3:00 min for the CP pulses including a 2D dual‐echo GRE prescan (TE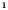 = 3.06 ms, TE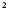 = 5.10 ms, TA = 60 s) at the same resolution for $B_{0}$ field map and coil sensitivity calculation [27]. In order to simulate the SE and STE signals of this experiment, $B_{1}^{+}$ (4 mm iso) and $B_{0}$ maps (3 mm iso) were acquired with the 3DREAM and dual‐echo GRE sequences.

The slice profiles of multiband CP and GRAPE pulses were measured with a spin‐echo spiral sequence with two spiral segments and in‐plane acceleration R = 3. We used additional Cartesian phase encoding in the slice direction to encode a FOV of 3.2 mm with 16 phase encoding steps (resolution: 0.2 mm). The slice profiles were measured for the spin‐echo signal after application of the multiband CP and GRAPE excitation and refocusing pulses with the same orientations as in the DWI acquisitions. The acquisition time was 3:44 min for the GRAPE pulses and 3:28 min for the CP pulses, the in‐plane resolution was 5 mm, and the spiral readout duration was 2 ms.

The performance of the transverse GRAPE pulses with respect to different slice orientations was investigated by acquiring data (30 volumes, b = 0 s/mm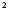) from the second subject at five distinct slice orientations, spanning a tilting range of 40° from the transverse toward the coronal plane.

### 2.3 Image Reconstruction & Processing

All EPI data were reconstructed with the vendor's slice‐GRAPPA reconstruction. The spiral data were reconstructed with an automated imaging pipeline [28]. The BART “pics” [29] command was used for slice profile measurements as no off‐resonance correction was needed due to the short spiral readout. The flip angle measurements were reconstructed with MatMRI [30, 31], which includes off‐resonance correction. All spiral k‐space trajectories were corrected for gradient imperfections using the gradient impulse response function [32].

All datasets except for the slice profile datasets were denoised in the magnitude domain [33, 34] using MRtrix3 [35]. All volumes in a dataset were jointly denoised using the smallest isotropic patch size that exceeds the number of volumes in the dataset, which was five for all datasets. The datasets were corrected for Gibbs‐Ringing [36] with MRtrix3, distortion‐ (only EPI) and motion‐corrected with FSL “eddy” [37, 38, 39] and registered to the midway space of CP and GRAPE TRA measurements with FSL “FLIRT” [40]. The SNR was estimated by dividing denoised mean $T_{2}$‐weighted and mean diffusion‐weighted images by the noise map obtained from the denoising step and afterwards applying the warp fields obtained from distortion and motion correction. Brain masks were calculated from 0.8 mm isotropic MPRAGE images (TA = 3:19 min) with ANTsPyNet [41] and registered to the diffusion space with FLIRT.

A diffusion tensor imaging (DTI) fit was performed with MRtrix3 [35, 42] and a neurite orientation dispersion and density imaging (NODDI) fit was performed with the accelerated microstructure imaging via convex optimization (AMICO) [43].

Flip angle ratios of the refocusing pulses were calculated from the SE and STE signals $S_{\text{SE}}$ and $S_{\text{STE}}$ with the equation [22]:

(1) $$r=\alpha /\alpha_{\text{nom}}=\arccos\left(\frac{S_{\text{STE}}e^{\frac{\text{TM}}{T_{1}}}}{S_{\text{SE}}}\right)/\alpha_{\text{nom}},$$

where TM = 100 ms is the mixing time and T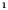 = 1500 ms is assumed for the whole brain. The mixing time was minimized within the SAR limits to reduce the bias due to varying T1 values across the brain [23]. As Equation (1) does not allow for separation of flip angles $90^{\circ }+\delta$ and $90^{\circ }-\delta$ (for arbitrary δ), the correct flip angle is determined by minimizing the variance for all $2^{12}$ permutations (two options, 12 nominal flip angles). Only the four pairs of SE/STE images with the highest SE signal were used for each voxel as proposed by Lutti et al. [24]. Voxels below a SE signal threshold of 10% of the 99th percentile of the SE signal and the top 5% voxels with the highest coefficient of variation were masked out and the flip angle ratio was interpolated from neighboring voxels using inverse distance weighting [24]. The final flip angle ratio maps were filtered with a 2 mm FWHM Gaussian kernel and registered to the diffusion space.

### 2.4 Pulse Simulations

Bloch simulations were conducted for all multiband pulses using the $B_{0}$ and $B_{1}^{+}$ maps of the measured subjects. The simulations were performed for the same matrix size as the $B_{1}^{+}$ maps with 4 mm isotropic voxels. For each 4 mm voxel, the multiband pulse with the smallest distance to the voxel center was selected for simulation using the same slice positions as in the optimization. The magnetization vector was then calculated at 31 equidistant positions along a 9 mm line in the slice direction centered around the voxel position such that the slice profile of the magnetization is obtained at a resolution of 0.3 mm. For this purpose, the $B_{1}^{+}$ and $B_{0}+$ maps were linearly interpolated along this line. Slice rewinder gradients after the excitation pulse and crusher gradients before and after the refocusing pulse were included in the simulations, relaxation effects were neglected. In order to correctly account for the effects of the crusher gradients, the magnetization was averaged over 100 equally spaced spins per position. All simulations were performed for the successive application of both pulses and separately for excitation and refocusing pulses to investigate their individual performance.

The signal in each 4 mm voxel was calculated by integrating the transverse magnetization over all positions along the slice profile. In order to calculate the signal for the individual simulation of the refocusing pulse, the simulation was started with purely transverse magnetization. For a better visualization of the slice profiles, the simulations were repeated for three distinct voxels in one subject by evaluating the magnetization at 180 positions (300 averaged spins per position) leading to a slice profile resolution of 0.05 mm. The same simulations were also performed for the transverse GRAPE pulses with different slice orientations by linearly interpolating the $B_{1}^{+}$ and $B_{0}$ maps according to the respective slice orientation.

In order to compare the measured flip angle ratios of the refocusing pulses to the simulations, the SE and STE signals were simulated for the same nominal flip angles as in the measurements. The SE and STE signal were then calculated as the integral over the simulated slice profile and the flip angle ratio map was calculated with the same method as for the measurements using Equation (1).

The spatial‐spectral response of the pair of CP or GRAPE excitation and refocusing pulses was obtained for three distinct voxels by simulating the signal along a 15 mm line in slice direction (301 positions, 100 averaged spins per position) for 33 different offset frequencies between 300 and 1300 Hz.

## 3 Results

The multiband pulse parameters after the optimization are listed in Table 1. The final bandwidths of the GRAPE pulses were slightly lower compared to the target bandwidth. An example of optimized singleband and multiband pulses for one slice group is shown in Figure 1 along with the optimized gradients. The RF magnitude of the optimized pulses does not differ strongly from sinc shapes, but the pulses show differing phase patterns. The optimized gradients of the excitation and refocusing pulses exhibit a similar shape.

**TABLE 1:** RF pulse parameters of CP, GRAPE TRA (transverse slice orientation), and GRAPE SAG (sagittal slice orientation) excitation and refocusing pulses.

Pulse	Dur (ms)	BW (Hz)	TBP	Energy (mJ)
*Excitation*				
CP	6	793	4.76	585
GRAPE SAG	6	654	3.93	443
GRAPE TRA	6	664	3.98	519
*Refocusing*				
CP	14	362	5.07	1379
GRAPE SAG	8	342	2.73	995
GRAPE TRA	8	330	2.64	999

*Note:* The pulse energies of CP pulses are averaged over all subjects, which have different reference voltages. The pulse energies of the GRAPE pulses are identical for each subject as their voltage is not scaled.

Abbreviations: BW, bandwidth; Dur, duration; TBP, time‐bandwidth product.

**FIGURE 1:**
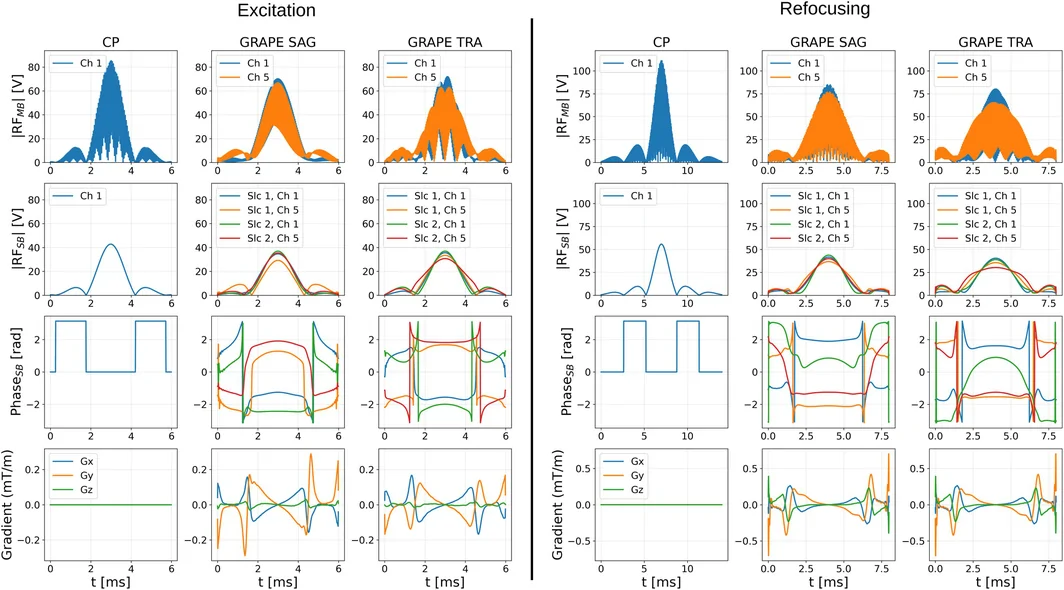
The figure shows the RF magnitude (channels 1 and 5) of optimized excitation and refocusing multiband pTx pulses (MB=2) for transverse (GRAPE TRA) and sagittal slices (GRAPE SAG) for one example slice group along with standard CP pulses (top row). The multiband pulses are composed of two singleband pulses, which are shown in the second (RF magnitude) and third (RF phase) row. The fourth row shows the optimized gradients, which are identical for all slice groups. For better visualization, the constant slice gradient is not plotted. The CP pulse shapes are the same for each channel/slice and are plotted for a reference voltage of 270 V. The phase of the multiband pulses is rapidly varying and not shown.

Figures 2 and S7 show the simulated signal after excitation and refocusing pulses obtained from the Bloch simulation. The overall signal homogeneity is improved for both GRAPE pulses compared to CP, which is reflected in a lower standard deviation of the simulated signal across the brain. The overall signal is higher for the GRAPE SAG pulses compared to the GRAPE TRA and CP pulses. Across all subjects, the GRAPE SAG pulses have the highest mean and lowest standard deviation, followed by GRAPE TRA and CP pulses (cf. Table S1). In the cerebellum, the signal is higher for the transverse GRAPE pulses compared to the sagittal GRAPE pulses and lowest for the CP pulses. The simulated slice profiles of three voxels in the superior part of the brain (voxel 1), in the cerebellum (voxel 2) and in the inferior frontal lobe (voxel 3) are shown on the right of Figure 2. The slice profiles of voxels 1 and 2 are mostly undistorted for all pulses, but the magnitude of the transverse magnetization is strongly reduced in the cerebellar voxel especially for the CP pulses. The slice profile in voxel three, located in the inferior frontal lobe, is shifted for all pulses due to the large off‐resonance frequency in this region.

**FIGURE 2:**
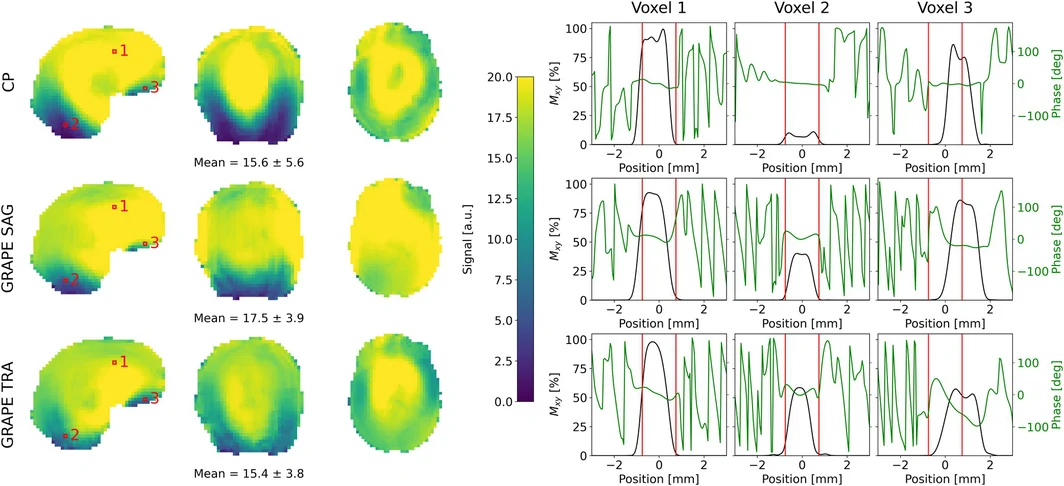
Results of the Bloch simulations after successive application of excitation and refocusing pulses. Left: Simulated signal for CP, GRAPE SAG and GRAPE TRA pulses for subject 5. Right: Simulated transverse magnetization along the slice direction (“slice profiles”) for three distinct voxel positions indicated by a red square in the signal maps. The voxels were selected to represent brain regions with varying degrees of $B_{1}^{+}$ inhomogeneity and off‐resonance frequency. The off‐resonance frequencies were 74 Hz (voxel 1), 15 Hz (voxel 2) and −220 Hz (voxel 3). The magnitude of the transverse magnetization is shown in black, the phase is shown in green and the nominal slice thickness is indicated by the red lines.

These results are confirmed by the slice profile measurement shown in Figure 3. Shifted slice profiles are observed for voxels located in the inferior frontal lobe shown on the right. The shifted slice profiles lead to significant slice bending and slice narrowing due to mismatches between the bandwidths of excitation and refocusing pulses in this region. The slice bending is slightly more pronounced for the GRAPE pulses compared to CP pulses. In contrast, slice bending is not observed in slices where the off‐resonances are less severe as shown on the left in Figure 3.

**FIGURE 3:**
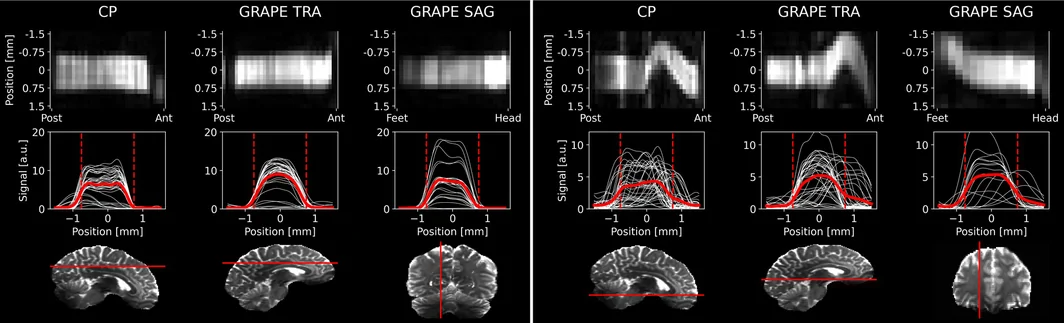
The figure shows slice profiles of the multiband pulses for subject 5 at two distinct slice positions acquired with the same orientation as the DWI acquisitions. Slices on the left are located in regions with low off‐resonance frequencies, while slices on the right contain large off‐resonance frequencies in the inferior frontal lobe. The top row shows the slices measured with CP and GRAPE pulses. In the second row, the slice profiles at all positions along the slice (posterior to anterior for CP and GRAPE TRA, feet to head for GRAPE SAG) are displayed in white. The mean slice profile is shown in red and the target slice thickness is indicated by the dashed red lines. The third row shows the position of the slice within the brain.

The patterns of the simulated and measured flip angle ratios of the refocusing pulse shown in Figure 4 are in good agreement. Significant overflipping is observed in the center of the brain for CP pulses, while underflipping is observed in the cerebellum. In contrast, the flip angle ratios of the GRAPE pulses show a more homogeneous distribution across the brain. Mean and standard deviation values across the brain indicate that the sagittal GRAPE pulses lead to the most homogeneous flip angle distribution, followed by the transverse GRAPE pulses and CP pulses. The measured flip angle ratio in the cerebellum is highest for the transverse GRAPE pulses. Histograms in Figure S9 show slightly higher flip angle ratios in the measurements compared to the simulations, which might be caused by T1 values deviating from the assumption or by low SNR voxels.

**FIGURE 4:**
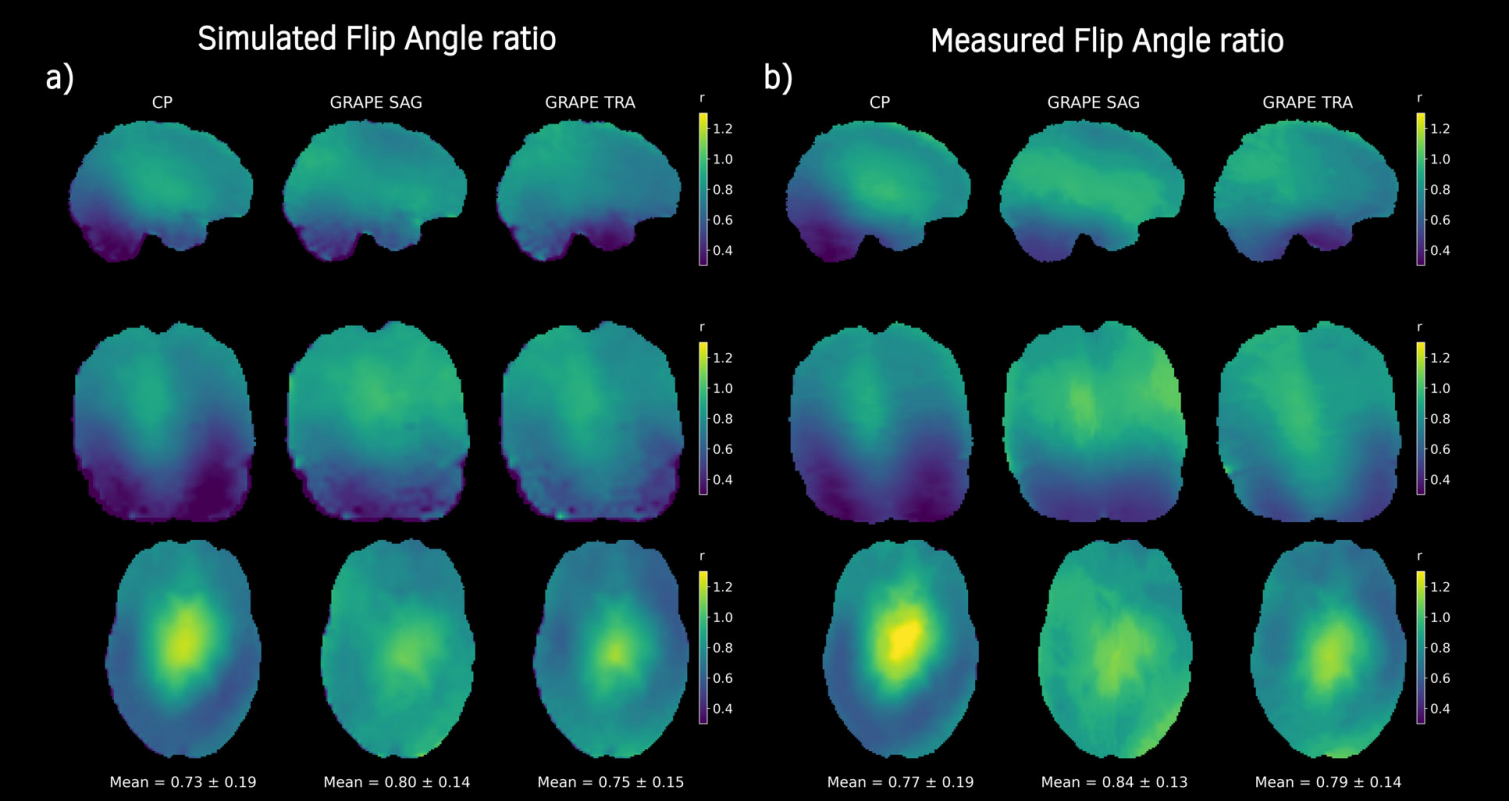
Simulated (a) and measured (b) flip angle ratio of CP and GRAPE refocusing pulses for subject 5. Histograms of simulated and measured flip angle ratios can be found in Figure S9.

In Figure 5 T2‐weighted images for three different head sizes are compared. It is visible, that the signal towards the lower cerebellum decreases with increasing head size for all pulses. Compared to the CP pulses, the signal in the cerebellum is increased for both GRAPE pulses across all head sizes. Signal voids in the temporal lobes are observed for transverse GRAPE pulses, when the head size is large. As signal homogenization is most challenging for large head sizes, all subsequent results are shown for subject 5, who had the largest head size. The respective results for subjects one and two (small and medium head sizes) are shown in Figures S1 and S2.

**FIGURE 5:**
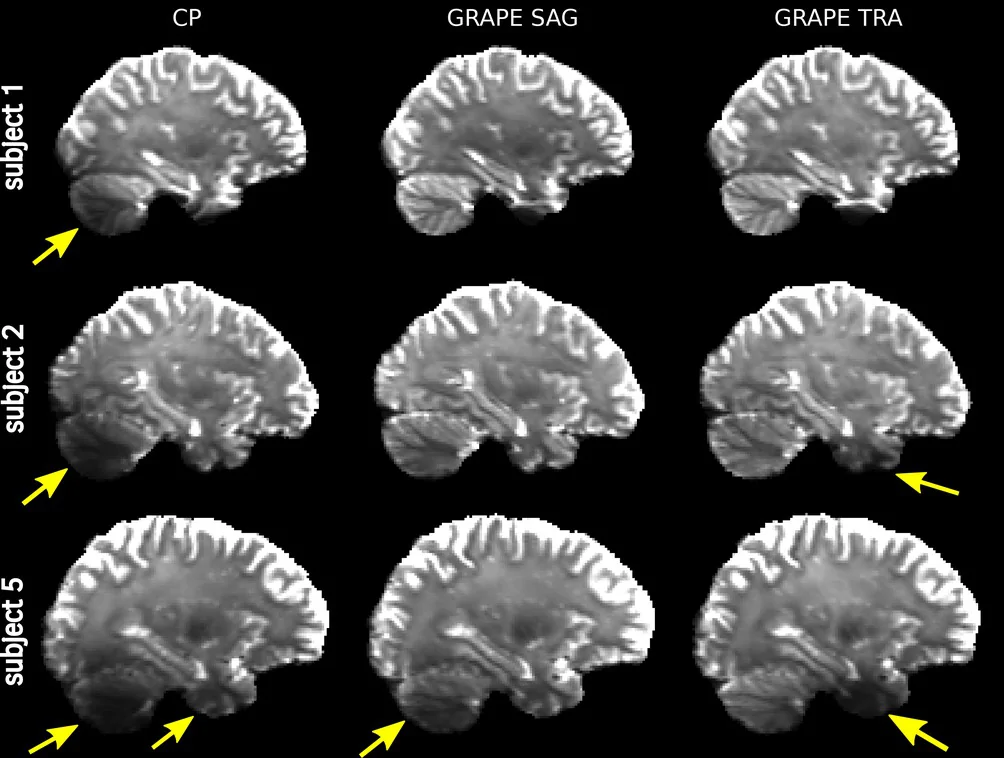
Comparison of $T_{2}$‐weighted images from subjects with small (subject 1), medium (subject 2), and large (subject 5) head sizes. Arrows mark areas with low signal. Similar slice positions were chosen for all three subjects.

Figure 6 shows mean $T_{2}$‐weighted and diffusion‐weighted images acquired with CP and GRAPE pulses. GRAPE pulses show enhanced signal in the cerebellum and higher overall homogeneity compared to CP pulses in both $T_{2}$‐weighted and diffusion‐weighted images. The signal in the cerebellum is higher for transverse GRAPE pulses compared to sagittal GRAPE pulses, which is consistent with the simulation results. However, reduced signal is observed in the frontal part of the temporal lobe and in the inferior frontal lobe. These are both regions where off‐resonances are typically large. The mean estimated SNR in the brain of both $T_{2}$‐weighted and diffusion‐weighted images is highest for sagittal GRAPE pulses, followed by transverse GRAPE pulses and lowest for CP pulses (cf. Figure 6). The results for the 1.05 mm resolution acquisitions are shown in Figures S3 and S4. For a multiband factor of two the improvements in homogeneity and SNR are similar to the 1.5 mm resolution acquisitions. For a multiband factor of three, the performance of the GRAPE pulses decreases due to the more restrictive SAR constraints.

**FIGURE 6:**
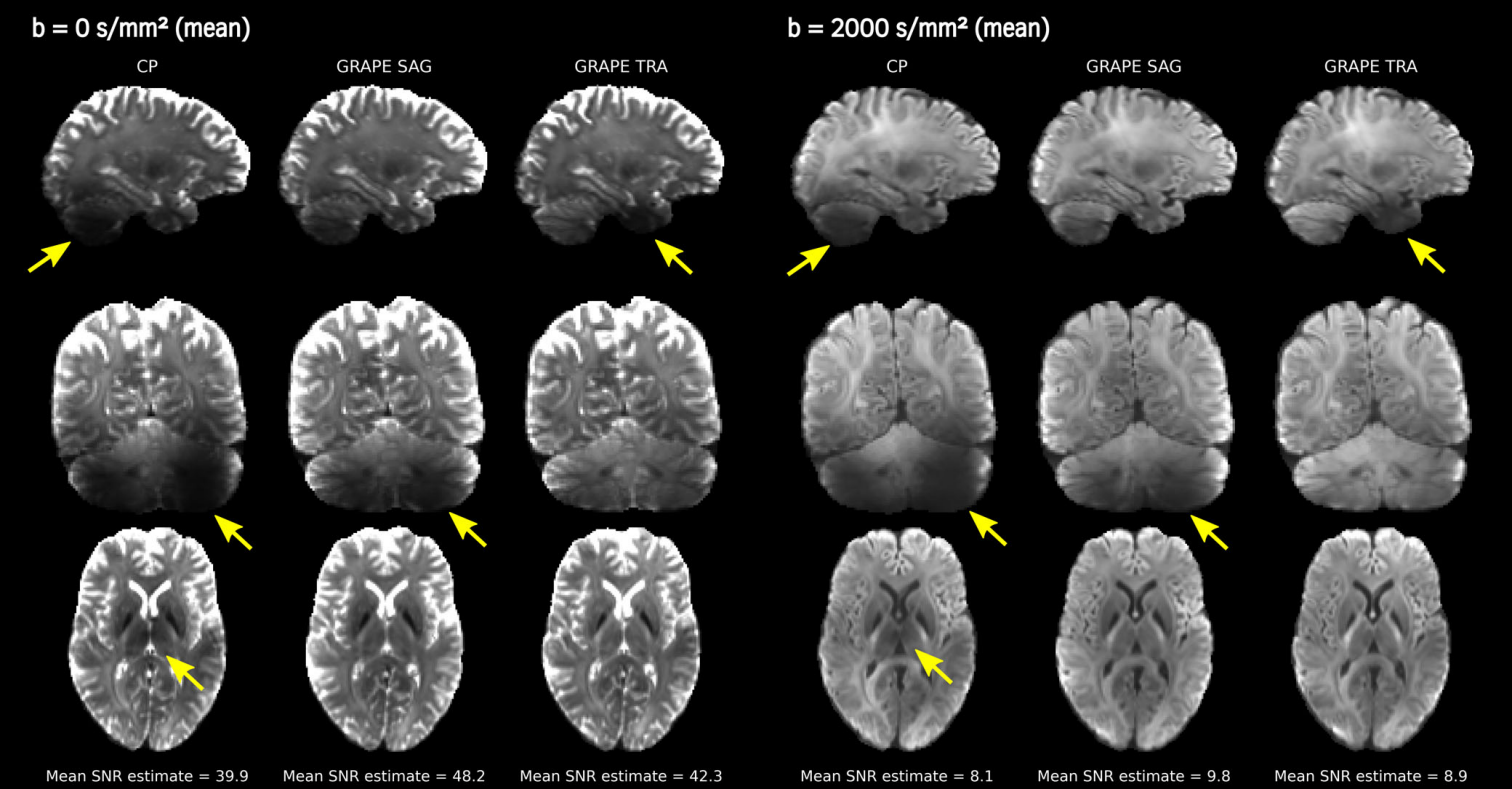
Mean $T_{2}$‐weighted (b = 0 s/mm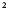) and diffusion‐weighted (b = 2000 s/mm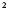) images for CP, GRAPE TRA and GRAPE SAG pulses for subject 5. Arrows mark areas with low signal in $T_{2}$‐weighted images. The mean estimated SNR values in the brain are listed below each column.

Figures 7 and 8 show the results of the DTI and NODDI fits for subject 5. Improved fit results in the cerebellum are observed in the fractional anisotropy maps for the GRAPE pulses compared to CP pulses. As the head size is large for this subject, degraded fit results are visible in the temporal lobe for GRAPE TRA pulses, as the signal is low in that region. The results of the NODDI fit show a stronger dependence on the signal level compared to the DTI fit. The NODDI fit fails in low‐SNR regions resulting in unrealistically high values for the orientation dispersion index (ODI) and neurite density index (NDI). The signal enhancement due to the GRAPE pulses visible in the raw diffusion‐weighted images translates directly to improved NODDI maps. For the 1.05 mm resolution datasets in Figures S3 and S4, the DTI fit shows reasonable results despite the lower SNR, while the NODDI fit quality is significantly reduced in large parts of the brain for all pulses. This is more pronounced for the GRAPE pulses with multiband factor three, where the SNR is lowest.

**FIGURE 7:**
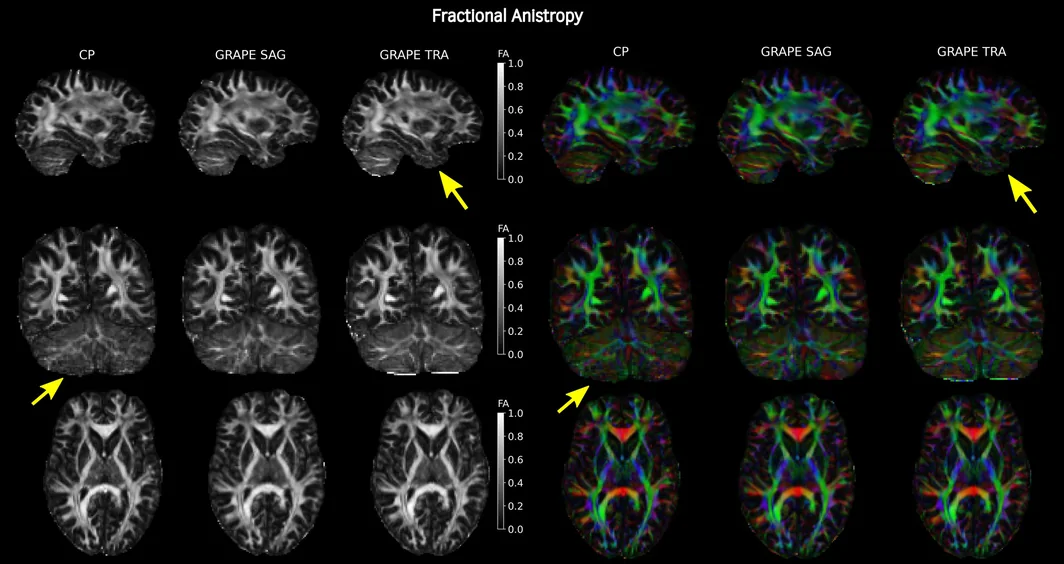
Results of the DTI fit for subject 5. Left: Fractional anisotropy maps for the three pulses: CP, GRAPE sagittal, and GRAPE transversal. Right: Color‐coded fractional anisotropy maps for the same acquisitions (red = left–right, green = anterior–posterior, blue = superior–inferior fiber orientation). Arrows mark regions where the DTI fit is unreliable due to low SNR.

**FIGURE 8:**
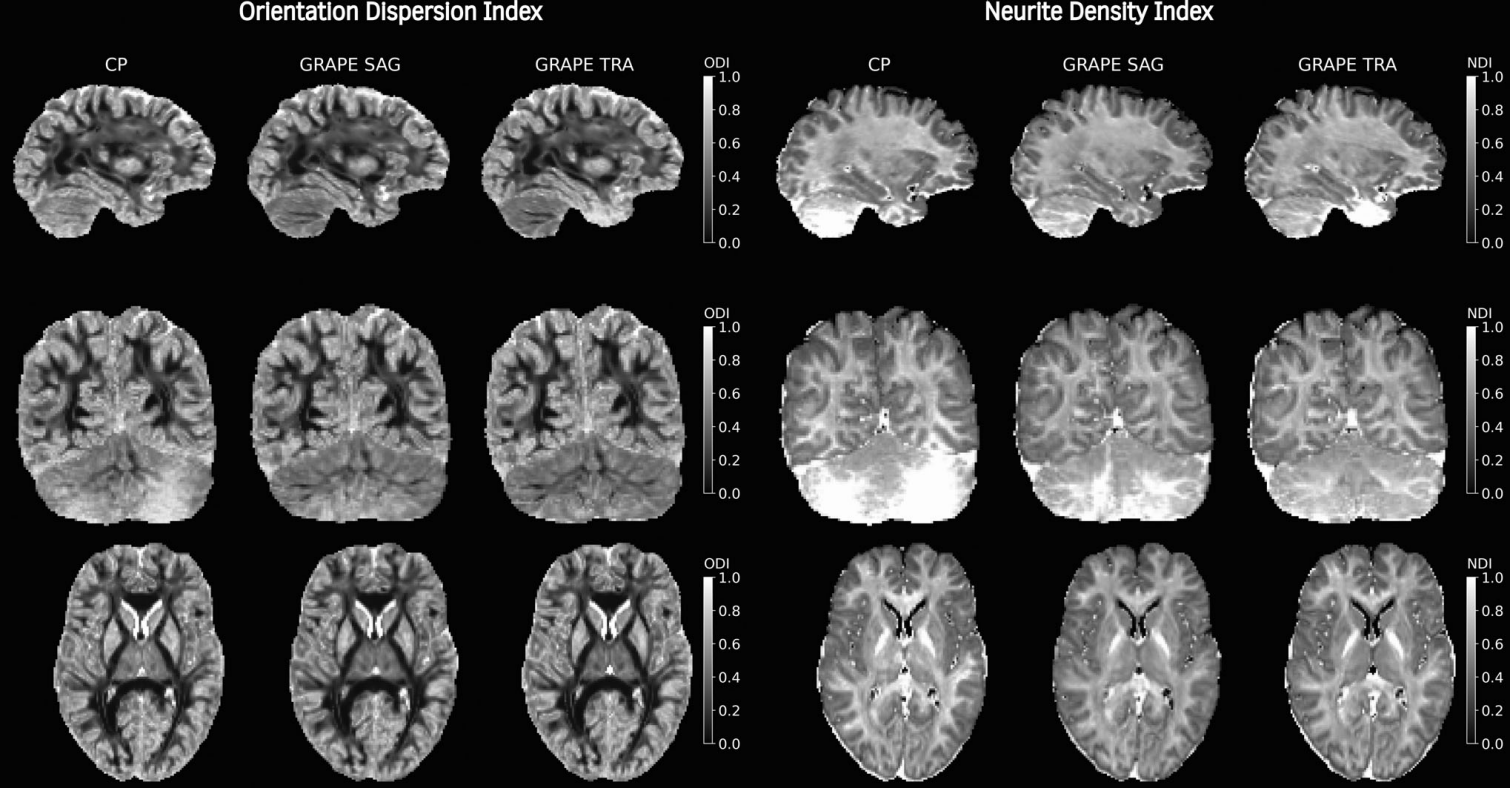
Results of the NODDI fit for subject 5. Orientation dispersion index (ODI; left) and neurite density index (NDI; right) maps are shown for the three pulses: CP, GRAPE sagittal, and GRAPE transversal.

The performance of the transverse GRAPE pulses across different slice orientations is shown in Figure 9. Signal homogeneity remained largely consistent with the exception of the inferior frontal lobe, where greater signal loss was observed at larger negative tilt angles. Positive tilts even showed slightly improved signal in the inferior frontal lobe compared to the transverse orientation.

**FIGURE 9:**
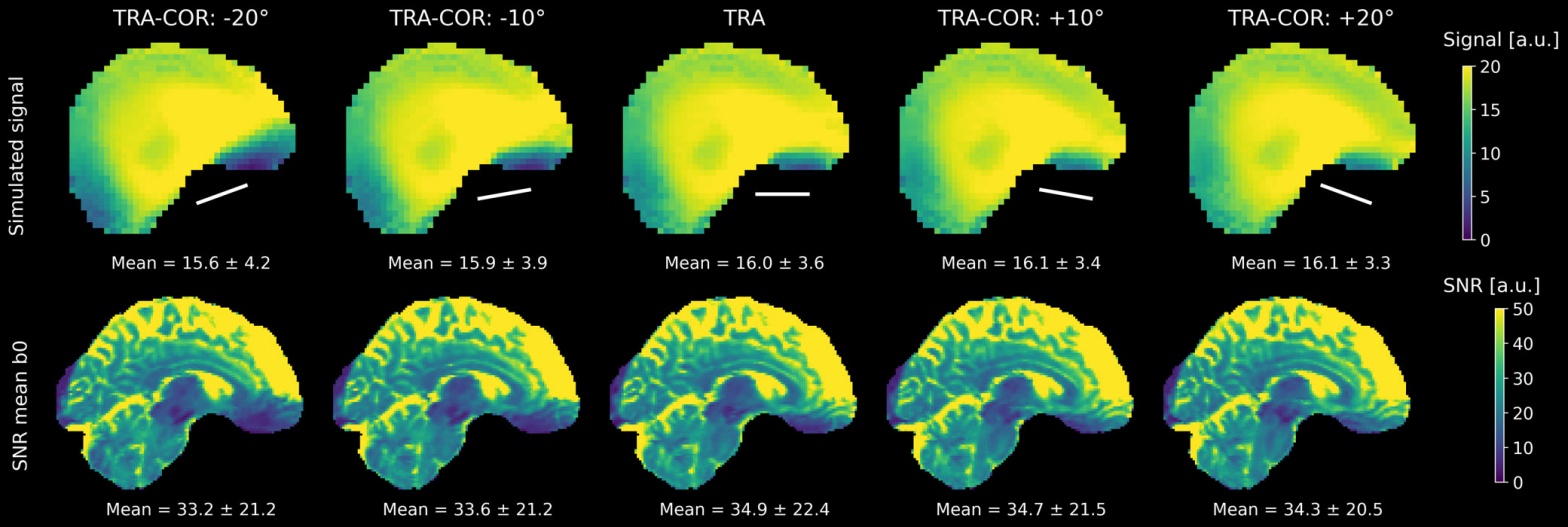
Simulated signal maps (top row) and measured SNR estimates of T2‐weighted images (bottom row) for the transverse GRAPE pulses using different slice orientations. The slice orientation is indicated by the white lines in the top row.

## 4 Discussion

We showed that the optimized universal multiband GRAPE pulses improve overall signal homogeneity and enhance signal intensity in whole‐brain DWI acquisitions at 1.5 and 1.05 mm resolution compared to standard CP pulses. The pulses particularly increase signal in the cerebellum, which is a region with typically low signal in CP mode at 7T. The better signal homogeneity translates to improved fit results for diffusion models as shown for DTI and NODDI. More complex diffusion models like NODDI benefit more from the improved signal in the cerebellum compared to the simple DTI model, as the number of fit parameters is larger, which makes the fit more susceptible to low SNR.

One challenge in the optimization of the slice‐selective universal pulses is the slice position, which can substantially change between different subjects and sessions. We accounted for this by adding an exponential weighting to the optimization and showed that this weighting allows for some variation in slice positioning. We investigated GRAPE pulses with sagittal and transverse slice orientations. While the sagittal pulses lead to higher overall signal, they still show reduced signal in the cerebellum especially in large heads. The transverse pulses result in higher signal in the cerebellum, but lead to signal voids in the temporal and inferior frontal lobe of large heads, where off‐resonance frequencies are typically high.

We chose to optimize the pTx pulses for purely sagittal and transverse slices rather than oblique transverse slices, although the number of slices and the acquisition time for a whole‐brain acquisition increases by approximately 10% for two reasons: First, purely sagittal and transverse orientations allow for more consistent and reproducible placement of the imaging volume, as well‐defined reference points can be used for positioning (top and center of the brain). Second, in the oblique transverse orientation lower slices in the stack contain the cerebellum, the brainstem and the inferior frontal lobe. These are areas with strongly diverging off‐resonance frequencies caused by air cavities. A large range of off‐resonance frequencies within the same slice is unfavorable for the optimization and leads to signal voids in the resulting images [12].

GRAPE pulses perform significantly better than CP pulses across different head sizes. However, signal in the lower cerebellum is still reduced compared to other brain regions especially in large heads. Optimization of GRAPE pulses on a subpopulation containing only larger heads could potentially improve the performance in this region [44, 45]. A second strategy is to include an additional spatial weighting in the optimization, which increases towards the cerebellum. First results obtained with such a weighting are shown in Figure S5. Simulation and measurement both indicate a slight improvement in the cerebellum. Increasing this weighting further is possible, but might lead to decreased performance in other brain regions. Another approach to improve pulse performance is a joint design of the multiband pulses within one slice group, which might allow for a more efficient power deposition [46]. Lastly, a simple scaling of the RF voltage of the GRAPE pulses with the reference voltage of the subject was shown to improve pulse performance [47]. This scaling is only possible if it does not lead to a violation of the SAR constraints.

The main limitation in the optimization of the GRAPE pulses is SAR restrictions. As different SAR limits apply for pTx mode (10 W/kg) and for CP mode (20 W/kg) in first‐level mode, the GRAPE pulses can only use half of the maximum possible SAR compared to CP pulses. This effectively limits the achievable bandwidth and TBP of the GRAPE pulses, which is reflected in the lower bandwidths compared to CP pulses. We showed that CP and GRAPE pulses have similar slice profiles and observed significant slice bending for both CP and GRAPE in off‐resonant regions. Slice bending cannot be sufficiently corrected through pulse optimization in regions with large nonlinear $B_{0}$ gradients, as the achievable correction is limited by the transmit coil geometry and the available degrees of freedom. While a larger pulse bandwidth would reduce slice bending, simulations showed that the pulse performance significantly decreases using the same SAR constraints. SAR limitations also led to reduced performance of GRAPE pulses with multiband factor three. A comparison with vendor‐provided RF shimming was impossible for the given sequence and CP pulse parameters, as the SAR limits were exceeded.

In order to maximize the SAR constraints in the optimization, fat suppression pulses were disabled. The fat unfocusing approach [18] was used by adjusting excitation and refocusing bandwidths such that the fat excitation and refocusing slices do not overlap. We did not observe additional fat artifacts compared to a sequence where fat suppression pulses were enabled. We validated the fat suppression in simulations of the spatial‐spectral response, which is shown for three voxels in Supporting Figure S6.

One potential solution to further decrease the SAR of the GRAPE pulses would be the integration of the VERSE concept [48] into the optimization. This requires a simultaneous optimization of the pulse and the slice gradient by defining a target magnetization along an additional slice dimension, which increases the complexity of the optimization. Additionally, the varying bandwidth of VERSE pulses is difficult to combine with the fat unfocusing approach and additional fat suppression pulses might be required.

On the other hand, peak voltage constraints were not limiting the optimization, as the peak voltages of the optimized pulses were well below the maximum allowed voltage. Therefore, the peak voltage constraint was set conservatively to 95 V per channel, as this is straightforward to implement and allows for full parallelization of the slice‐specific pulse optimization while not adversely affecting the pulse design.

We observed that the pulse performance of the refocusing pulse is more crucial for the overall signal homogeneity compared to the excitation pulse. This is visualized in Figure S7, where the overall signal pattern is similar when simulating just the refocusing pulse or when simulating both pulses. The refocusing pulse therefore determines the homogeneity of the signal, while the performance of the excitation pulse mainly defines the overall signal level. Therefore, relaxed SAR constraints for the refocusing pulse and stronger constraints for the excitation pulse could potentially further improve the signal homogeneity.

As all EPI scans were GRAPPA and multiband accelerated, they require ACS prescans with singleband pulses. Using different RF pulses for the ACS scans and the image acquisition may lead to inconsistencies between the two datasets due to differences in slice profiles, coil sensitivity weighting, and ghosting artifacts, which may result in subtle artifacts [49]. However, no differences were observed when using singleband pTx pulses optimized for the whole brain or CP pulses for ACS scans (Figure S8).

Optimization of the presented pulses on a high performance cluster (72 CPU cores, 3 TB memory) took around five days to complete. As the pulses were designed as universal pulses, the optimization only has to be repeated if the pulse parameters, the database or the RF coil changes. Changing the number of slices is possible without repeating the optimization by recombining a subset of the optimized singleband pulses and exploiting our conservative peak voltage limit. Adjusting the slice thickness of the pTx pulses is theoretically possible by adjusting the slice gradient as the $B_{1}^{+}$ distribution is typically smooth and both the exponential weighting and the variation in the pulse design database create additional robustness against variation in slice position. Combining the universal pulse approach with the slab‐wise approach proposed by Wu et al. [50] would allow for a reduction of the number of pulse shapes, which reduces both memory demands of the optimization and preparation time of the pulses on the scanner. Additionally, it would allow for using slice‐specific singleband pulses in ACS scans, if the number of pulse shapes is reduced sufficiently.

## 5 Conclusion

Universal multiband GRAPE pTx pulses substantially improve signal homogeneity and SNR of whole‐brain diffusion MRI at 7T compared to standard CP mode, particularly in the cerebellum. The improved signal translates into more reliable diffusion model fitting, especially for complex models such as NODDI that are more susceptible to low SNR. Among the two investigated slice orientations, sagittal GRAPE pulses provided the highest overall signal and homogeneity, while transverse GRAPE pulses yielded better cerebellar coverage at the cost of increased signal loss in off‐resonant frontal regions. The main limitation in the pulse optimization is SAR constraints, which limit the achievable pulse bandwidth resulting in residual slice bending in regions with strong off‐resonance. As the pulses are designed as universal pulses, no individual calibration scans are required, making the pulses practical for routine use.

## Funding

This work received financial support from the Horizon 2020 Framework Programme under grant agreement 885876 (AROMA) and through the German Federal Ministry of Education and Research (BMBF; funding code 01ED2109A) as part of the SCAIFIELD project under the aegis of the EU Joint Programme—Neurodegenerative Disease Research (JPND) (https://www.jpnd.eu).

## Conflicts of Interest

The authors declare no conflicts of interest.

## Supporting information




**Table S1:** Mean and standard deviation of the SNR (b = 0 s/mm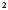 and b = 2000 s/mm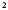) and the simulated signal after excitation and refocusing pulses across all brain voxels are listed for all five subjects.
**Figure S1:** Results corresponding to Figures 6–8 of the main manuscript, shown here for subject 1, who has a small head size. Mean T2‐weighted images (b = 0 s/mm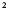), mean diffusion‐weighted images (b = 2000 s/mm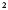), (colored) fractional anisotropy maps, orientation dispersion index maps and neurite density index maps are shown for CP, GRAPE TRA, and GRAPE SAG pulses.
**Figure S2:** Results corresponding to Figures 6–8 of the main manuscript, shown here for subject 2, who has a medium head size. Mean T2‐weighted images (b = 0 s/mm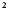), mean diffusion‐weighted images (b = 2000 s/mm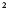), (colored) fractional anisotropy maps, orientation dispersion index maps and neurite density index maps are shown for CP, GRAPE TRA, and GRAPE SAG pulses.
**Figure S3:** Results obtained at 1.05 mm isotropic resolution, a multiband factor of two and in‐plane GRAPPA acceleration of three (subject 2). Mean T2‐weighted images (b = 0 s/mm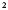), mean diffusion‐weighted images (b = 2000 s/mm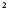), (colored) fractional anisotropy maps, orientation dispersion index maps and neurite density index maps are shown for CP, GRAPE TRA, and GRAPE SAG pulses.
**Figure S4:** Results obtained at 1.05 mm isotropic resolution and a multiband factor of three and in‐plane GRAPPA acceleration of three (subject 2). Mean T2‐weighted images (b = 0 s/mm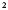), mean diffusion‐weighted images (b = 2000 s/mm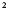), (colored) fractional anisotropy maps, orientation dispersion index maps and neurite density index maps are shown for CP, GRAPE TRA, and GRAPE SAG pulses.
**Figure S5:** Signal simulation and T2‐weighted images obtained with “non‐weighted” and “weighted” sagittal GRAPE pulses at 1.5 mm resolution. In the optimization of weighted sagittal pulses, an additional increasing weight towards the cerebellum was added. This slightly improves signal strength in the cerebellum, but also leads to slightly lower signal in the inferior frontal lobe.
**Figure S6:** Spatial‐spectral response for all three pulses after excitation and refocusing for the three distinct voxels marked in Figure 2 of the paper with off‐resonance frequencies 74 Hz (voxel 1), 15 Hz (voxel 2) and −220 Hz (voxel 3). The center of the water and fat resonances shifted by the respective off‐resonance frequency are marked by white and red solid lines.
**Figure S7:** Simulated signal and slice profiles for three example voxels after excitation, refocusing and successive application of both pulses (subject 5). The simulation of the refocusing pulse was initialized with purely transverse magnetization. Mean values and standard deviations across the brain are printed below each plot. Note that the refocusing CP pulse has a slightly thicker slice profile compared to the GRAPE pulses resulting in higher signal, if the simulation is initialized with purely transverse magnetization.
**Figure S8:** Raw images obtained for *b* values b = 0 s/mm^2 and b = 2000 s/mm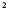 using CP or GRAPE singleband pulses in the autocalibration signal (ACS) scans. In both cases, transverse multiband GRAPE pulses were used for the imaging scans. The phase encoding direction was anterior‐posterior and no preprocessing was applied to the images. No visible difference is observed between the two scans.
**Figure S9:** Histograms of the simulated and measured flip angle ratio of subject 5 shown in Figure 4 of the paper for CP and both GRAPE pulses.

## Data Availability

The data that support the findings of this study are available from the corresponding author upon reasonable request.
