# Supplementary material for: Whole‐Brain Diffusion MRI at 7T With Universal Multiband GRAPE pTx Pulses
Source: Magn Reson Med. 2026 Sep 14;97(1):146–58. doi: 10.1002/mrm.70588 (PMC13746085; doi:10.1002/mrm.70588)
Supplement: Supplementary file 1 [file MRM-97-146-s001.docx]

Supporting Table S1: Mean and standard deviation of the SNR (b=0 and b=2000 s/mm^2^) and the simulated signal after excitation and refocusing pulses across all brain voxels are listed for all five subjects.

| Subject | SNR (b=0) [a.u.] | | | SNR (b=2000) [a.u.] | | | Simulated Signal [a.u.] | | |
| --- | --- | --- | --- | --- | --- | --- | --- | --- | --- |
|  | CP | GRAPE  SAG | GRAPE  TRA | CP | GRAPE  SAG | GRAPE  TRA | CP | GRAPE  SAG | GRAPE  TRA |
| 1 (f, 25) | 38.9 ± 32.3 | 47.3 ± 36.5 | 43.4 ± 32.7 | 8.1 ± 2.9 | 9.9 ± 3.7 | 9.3 ± 3.3 | 15.6 ± 5.1 | 17.9 ± 2.6 | 16.1 ± 3.2 |
| 2 (m, 25) | 40.0 ± 36.2 | 51.4 ± 40.7 | 44.5 ± 34.3 | 8.1 ± 3.0 | 10.3 ± 387 | 9.1 ± 3.1 | 14.8 ± 5.4 | 18.0 ± 3.2 | 16.0 ± 3.6 |
| 3 (m, 44) | 45.4 ± 40.9 | 55.6 ± 43.9 | 49.8 ± 41.2 | 8.5 ± 3.2 | 10.6 ± 3.9 | 9.5 ± 3.2 | 14.7 ± 5.4 | 17.8 ± 3.2 | 15.9 ± 3.7 |
| 4 (m, 28) | 35.8 ± 29.0 | 44.0 ± 33.7 | 39.5 ± 29.3 | 7.9 ± 2.8 | 9.3 ± 3.5 | 8.6 ± 2.9 | 14.7 ± 5.1 | 17.3 ± 3.4 | 15.4 ± 3.7 |
| 5 (m, 29) | 39.9 ± 35.4 | 48.2 ± 38.9 | 42.3 ± 32.8 | 8.1 ± 2.9 | 9.8 ± 3.6 | 9.0 ± 3.2 | 15.5 ± 5.5 | 17.5 ± 3.9 | 15.4 ± 3.8 |

Supporting Figure S1: Results corresponding to Figures 6-8 of the main manuscript, shown here for subject 1, who has a small head size. Mean T_2_-weighted images ($b=0 s/{mm}^{2}$), mean diffusion-weighted images ($b=2000 s/{mm}^{2}$), (colored) fractional anisotropy maps, orientation dispersion index maps and neurite density index maps are shown for CP, GRAPE TRA and GRAPE SAG pulses.

Supporting Figure S2: Results corresponding to Figures 6-8 of the main manuscript, shown here for subject 2, who has a medium head size. Mean T2-weighted images $(b=0 s/{mm}^{2})$, mean diffusion-weighted images $(b=2000 s/{mm}^{2})$, (colored) fractional anisotropy maps, orientation dispersion index maps and neurite density index maps are shown for CP, GRAPE TRA and GRAPE SAG pulses.

Supporting Figure S3: Results obtained at 1.05 mm isotropic resolution, a multiband factor of two and in-plane GRAPPA acceleration of three (subject 2). Mean T2-weighted images $(b=0 s/{mm}^{2})$, mean diffusion-weighted images $(b=2000 s/{mm}^{2})$, (colored) fractional anisotropy maps, orientation dispersion index maps and neurite density index maps are shown for CP, GRAPE TRA and GRAPE SAG pulses.

Supporting Figure S4: Results obtained at 1.05 mm isotropic resolution, a multiband factor of three and in-plane GRAPPA acceleration of three (subject 2). Mean T2-weighted images $(b=0 s/{mm}^{2})$, mean diffusion-weighted images $(b=2000 s/{mm}^{2})$, (colored) fractional anisotropy maps, orientation dispersion index maps and neurite density index maps are shown for CP, GRAPE TRA and GRAPE SAG pulses.

Supporting Figure S5: Signal simulation and T_2_-weighted images obtained with “non-weighted” and “weighted” sagittal GRAPE pulses at 1.5 mm resolution. In the optimization of weighted sagittal pulses, an additional increasing weight towards the cerebellum was added. This slightly improves signal strength in the cerebellum, but also leads to slightly lower signal in the inferior frontal lobe.

Supporting Figure S6: Spatial-spectral response for all three pulses after excitation and refocusing for the three distinct voxels marked in Figure 2 of the paper with off-resonance frequencies 74 Hz (voxel 1), 15 Hz (voxel 2) and -220 Hz (voxel 3). The center of the water and fat resonances shifted by the respective off-resonance frequency are marked by white and red solid lines.

Supporting Figure S7: Simulated signal and slice profiles for three example voxels after excitation, refocusing and successive application of both pulses (subject 5). The simulation of the refocusing pulse was initialized with purely transverse magnetization. Mean values and standard deviations across the brain are printed below each plot. Note that the refocusing CP pulse has a slightly thicker slice profile compared to the GRAPE pulses resulting in higher signal, if the simulation is initialized with purely transverse magnetization.

Supporting Figure S8: Raw images obtained for b-values $b=0 s/{mm}^{2}$ and $b=2000 s/{mm}^{2}$using CP or GRAPE singleband pulses in the autocalibration signal (ACS) scans. In both cases, transverse multiband GRAPE pulses were used for the imaging scans. The phase encoding direction was anterior-posterior and no preprocessing was applied to the images. No visible difference is observed between the two scans.

Supporting Figure S9: Histograms of the simulated and measured flip angle ratio of subject 5 shown in figure 4 of the paper for CP and both GRAPE pulses.
